# TEES 2.2: Biomedical Event Extraction for Diverse Corpora

**DOI:** 10.1186/1471-2105-16-S16-S4

**Published:** 2015-10-30

**Authors:** Jari Björne, Tapio Salakoski

**Affiliations:** 1 Department of Information Technology, University of Turku, Joukahaisenkatu 3-5, 20520 Turku, Finland; 2 Turku Centre for Computer Science (TUCS), Joukahaisenkatu 3-5, 20520 Turku, Finland

**Keywords:** BioNLP, event extraction, text mining

## Abstract

**Background:**

The Turku Event Extraction System (TEES) is a text mining program developed for the extraction of events, complex biomedical relationships, from scientific literature. Based on a graph-generation approach, the system detects events with the use of a rich feature set built via dependency parsing. The TEES system has achieved record performance in several of the shared tasks of its domain, and continues to be used in a variety of biomedical text mining tasks.

**Results:**

The TEES system was quickly adapted to the BioNLP'13 Shared Task in order to provide a public baseline for derived systems. An automated approach was developed for learning the underlying annotation rules of event type, allowing immediate adaptation to the various subtasks, and leading to a first place in four out of eight tasks. The system for the automated learning of annotation rules is further enhanced in this paper to the point of requiring no manual adaptation to any of the BioNLP'13 tasks. Further, the scikit-learn machine learning library is integrated into the system, bringing a wide variety of machine learning methods usable with TEES in addition to the default SVM. A scikit-learn ensemble method is also used to analyze the importances of the features in the TEES feature sets.

**Conclusions:**

The TEES system was introduced for the BioNLP'09 Shared Task and has since then demonstrated good performance in several other shared tasks. By applying the current TEES 2.2 system to multiple corpora from these past shared tasks an overarching analysis of the most promising methods and possible pitfalls in the evolving field of biomedical event extraction are presented.

## Introduction

Biomedical event extraction as a research field aims to develop annotations that can capture in detail the complicated relations between concepts in natural language texts. Compared to binary relation extraction, event extraction systems are more complicated, but through the use of nesting, typed and directed arguments and annotated trigger words can capture in more detail the semantics of the text.

In the development of biomedical event extraction the BioNLP Shared Task has been instrumental, providing a shared platform for comparison of diverse text mining methods. Originally organized in 2009, the BioNLP Shared Task has grown more varied in the following 2011 and 2013 iterations [1,2]. The original shared task used the NF-kB focused GENIA corpus, but in later years targets as diverse as epigenetics and bacteria-host interactions have been introduced. The 2013 task concerns "knowledge base construction", utilizing multiple domain corpora to drive the development of the kind of text mining systems required for automatically assisted database curation [3].

The Turku Event Extraction System (TEES) (http://jbjorne.github.com/TEES/) was developed originally for the 2009 BioNLP Shared Task but has since then grown into a generalized biomedical event extraction tool. It uses a graph representation to break the task of event extraction down into discrete, consecutive classification steps, using large feature sets and efficient support vector machines (SVM) to achieve good performance. The TEES system achieved the first place in the original BioNLP Shared Task, and first place in four out of eight domain tasks in both the 2011 and 2013 BioNLP Shared Tasks [4,5]. The TEES system was made available as an open source project in 2009, and has since then been applied for different event extraction tasks also by other research groups [6,7].

The BioNLP Shared tasks provide a record of the development of event extraction systems. In the 2009 task the original TEES 1.0 system achieved an F-score of 51.95%. In the similar 2011 GENIA task a best performance of 56.0% F-score was reached by team FAUST [8]. In the interim of these Shared Tasks the EventMine system of Miwa et al.[9] reached 56.00% on the original 2009 GENIA corpus. In 2012 Bui et al. [10] introduced a very computationally efficient system that learned automatically extraction rules from event templates. The GENIA corpus used in the 2013 BioNLP Shared Task has been drastically remodeled so a direct comparison with the earlier tasks is no longer meaningful.

In participating in the BioNLP 2013 Shared Task the TEES project aimed to improve the generalization of its event extraction approach, originally introduced in the 2011 task. The learning of event annotation rules was fully automated, based on a rule-based analysis of each task corpus. As an open source project TEES could potentially be useful also for other participants to expand and build on, but despite extensive work on the system, might be too complicated to easily apply. Therefore, the predictions of the TEES 2.1 system were also provided as open data, available for any interested participant, during the system development phase of the 2013 Share Task.

After the BioNLP 2013 Shared Task the automated annotation scheme learning system was finalized in the work described in this paper, leading to the TEES 2.2 system that is finally capable of processing any of the applicable BioNLP Shared Task corpora with no task-specific manual adaptation required. To help in analyzing the predictions of the system, a visualizer is also provided in the 2.2 release. As a major new direction for the system, an integration with the scikit-learn library is introduced, allowing the application of the vast variety of high-performance classifiers from this widely used machine learning library within the TEES system. An ensemble method provided by scikit-learn is used to analyze the feature sets used in TEES. To analyze the TEES 2.2 performance, the system is now applied to all the past BioNLP Shared Tasks, providing an overall picture of the relative complexity of the various corpora when approached with machine learning methods. This paper builds on and extends the BioNLP'13 workshop publication [11].

## Methods

### TEES overview

The Turku Event Extraction system utilizes a step-wise machine learning approach to detect complex text-bound graphs in biomedical domain natural language texts. The TEES approach is based on a generalized graph format, applicable for both events and binary relations. With this graph format, the complex task of event extraction is broken down into straightforward, consecutive graph node or edge classification tasks. A rich set of features is derived for each task from the graph format, leading to large datasets, that can however be efficiently handled by the SVM*^multiclass ^*support vector machine (http://svmlight.joachims.org/svm_multiclass.html) [12] when used with a linear kernel. The Turku Event Extraction System is described in detail in [13].

#### The graph format

TEES models all event and relation extraction as a task of graph generation. Named entities and event triggers form the nodes of the graph, and each node is bound to a single text token, the *syntactic head *of the text span covered by the node. Binary relations, which can be typed and directed, are edges that connect two nodes. Events are represented indirectly: The trigger word is a node, and event arguments are directed edges that connect the trigger word to other nodes. A single event thus consists of a trigger node and its set of outgoing argument edges. By modeling both events and relations as nodes and edges, TEES can process different kinds of semantic annotations with the same graph generation pipeline.

The graph is commonly stored in an Interaction XML file, the internal format of the TEES system, based on a generic, extensible XML representation developed to be applicable for diverse corpora [5,14,15]. A detailed description of the Interaction XML format is provided in the TEES online documentation (https://github.com/jbjorne/TEES/wiki/Interaction-XML). When TEES is applied to the BioNLP Shared Tasks, a built-in conversion system is used to turn the shared task files (txt/a1/a2) into interaction XML and vice versa. In this process, the BioNLP Shared Task equivalence annotation is also expanded into individual events.

**Figure 1:**
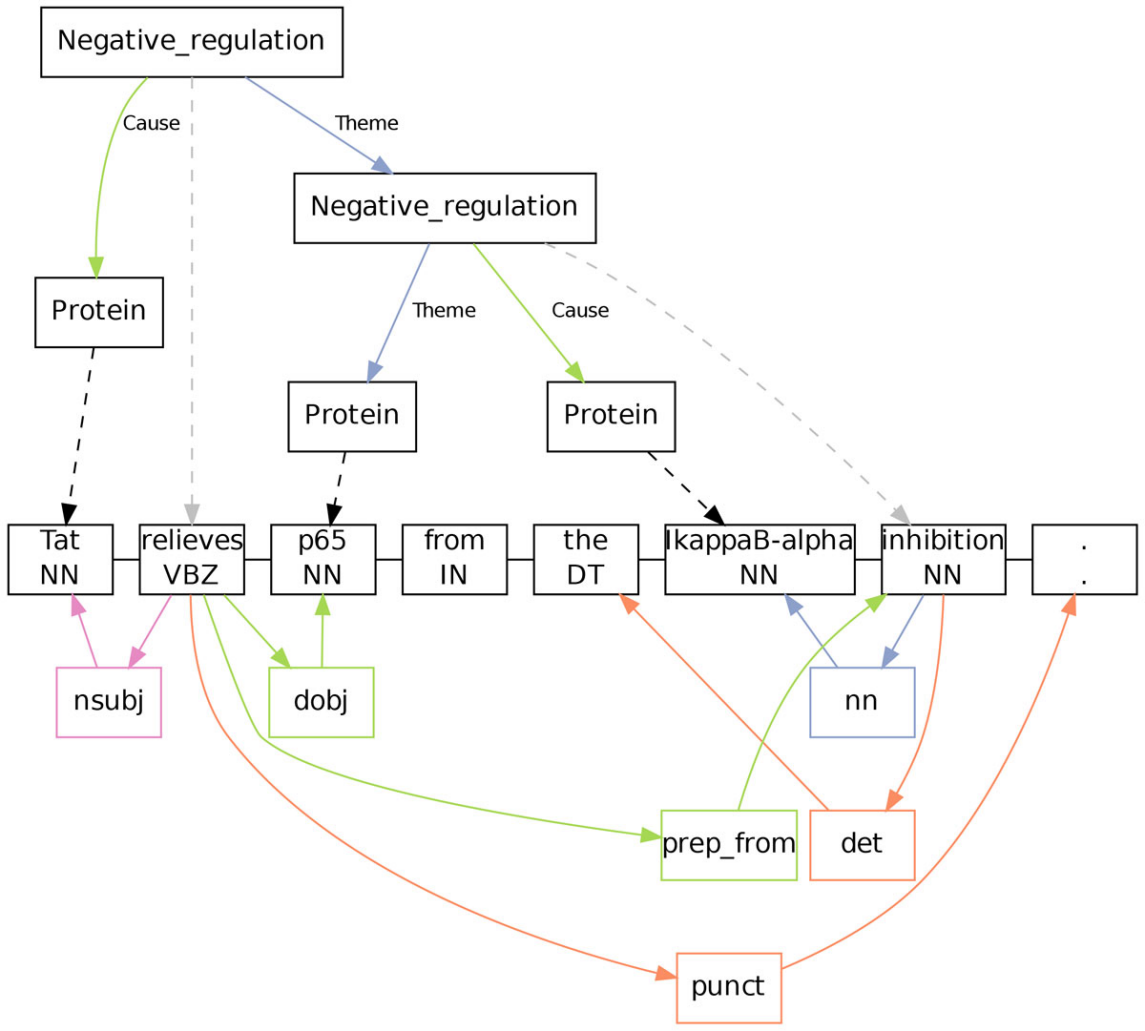
**The TEES event extraction process**. Preprocessing steps A-C can be omitted in the BioNLP Shared Tasks as corresponding data is provided by the organizers. The event extraction steps D-F are all independent SVM classification steps, with the trigger and edge detection steps being linked together by the recall adjustment parameter. (Figure adapted from Björne et. al [5].)

The TEES event extraction process is shown in Figure 1. The three primary phases of the system are the *entity, edge *and *unmerging *steps. A *recall adjustment *parameter that increases the amount of predicted entity nodes is determined experimentally against overall system performance, thus optimizing the entity detection step for the larger task of event extraction. The feature representations and basic approach of the system are largely unchanged from the 2011 entry [5].

In the BioNLP Shared Tasks the participants are given pre-parsed texts where named entities (protein and gene names) have already been marked. In real-world applications these preliminary steps can be performed using the TEES preprocessor. As in previous Shared Tasks, we used in this work the official tokenisations and the McCCJ parses converted into the collapsed CC-processed Stanford dependency scheme, provided by the organizers [16,17].

#### Entity detection

The first step in the TEES pipeline is the prediction of nodes, named entities and event triggers (See Figure 1C). In the BioNLP Shared Tasks, many of the named entities are already given. As with all TEES steps, the syntactic parse is the main source of features. As each node must be linked to a single syntactic token, TEES generates one classifiable example for each token (that is not already part of a named entity) in the text. With multiclass classification, this token is classified into one of the positive classes (e.g. *Phosphorylation, Regulation*) or as a negative. Overlapping nodes of different types are handled via merged classes, (e.g. *Phosphorylation-Regulation*) which are split into their components after classification. The entity detection step produces the nodes of the graph, and the next step is to find the relations that connect them.

#### Edge detection

Edges are the binary relations and event arguments that link together the nodes of the graph (See Figure 1D). When the nodes are known, edge detection proceeds by constructing one classifiable example for each (optionally directed) pair of nodes. As with nodes, edge examples are classified into a number of positive classes (e.g. *Theme *or *Cause*), or as negatives. As with nodes, overlapping edges of different types are handled with merged classes. The result of edge detection is a *merged *semantic network, which contains all the events, relations and arguments, but where overlapping events are merged together.

#### Unmerging

In event annotations, multiple events with different arguments can share the same trigger. In the TEES pipeline nodes (such as triggers) are predicted first, at a time when it cannot be said how many events will use them. The result is that when event arguments are predicted between these nodes, all overlapping events of the same type will be merged into a single node and its set of outgoing edges. The unmerging step addresses this issue: A classifiable example is generated for each node, for each valid set of outgoing edges, i.e. for each structure that can potentially be a valid event. The classifier classifies these into true events or negatives, and the final graph, with merged events "pulled apart" is constructed from these predictions (See Figure 1E). This graph can then straightforwardly be converted into the BioNLP Shared Task format.

Following unmerging, for those tasks where events can have modifiers, an additional modifier detection step can be performed.

### Automated Annotation Scheme Learning

The TEES 2.1 system, developed for the BioNLP 2013 Shared Task, introduced an automated annotation scheme learning system, automatically adapting the system for the annotation rules of different corpora. Further improved in the 2.2 version, this preprocessing tool generates an annotation scheme definition that the various classification steps (described in Section TEES overview) rely on.

If earlier versions of TEES were to be used with new corpora, task specific rules had to be defined by manually extending the program code. The most important function for such rules was to define the type and number of arguments for valid events of each annotation scheme. Thus, TEES could only be easily used to detect events similar to the ones in the shared task corpora for which corpus-specific code had been written.

In the current system, the event scheme and its constraints are learned automatically. By analyzing the full, known, annotated corpus (usually training and development sets) the system determines the annotation rules from the annotated events and relations. This learning system is fully deterministic and rule-based, and learns five kinds of annotation definitions:

**Entities **are nodes that cannot have outgoing edges. They usually represent the named entities of the annotation scheme. A node type is defined as an *entity*, if it does not have the "event" attribute set and if it never has outgoing edges.

**Relations **are the binary interactions of the annotation scheme. All interactions, both binary ones as well as event arguments are represented as interaction elements in the TEES graph format. An interaction type is defined as a *relation *if it does not have the "event" attribute set. A relation can be either directed or undirected, defined by the "directed" attribute of the interaction element.

**Events **are the BioNLP Shared Task style complex events, which consist of a trigger and multiple arguments. The annotation scheme learning system detects as event triggers all nodes that have outgoing edges, or that have the "event" attribute set. The types of these nodes define the types of the events. After event types are defined, the system iterates over all interactions in a document, and groups them in event instances. The maximum and minimum number of allowed arguments, for each event type, is updated from the argument counts of each event instance. The overall maximum and minimum number of arguments for each event type is updated from the limits of each argument type. The result is an *event *definition that defines the type of the event, the number of arguments it can have, and for each of its argument types, the valid minimum and maximum number.

**Modifiers **are the negation and speculation attributes defined in some BioNLP Shared Tasks, such as the GENIA tasks. If a node is seen having either of these modifiers, it is defined as a target for such modification in the learned annotation scheme.

**Targets **are the node types (entity and event trigger types) and edge types (relation and event argument types) that are the prediction targets for the corpus. In many of the BioNLP corpora, various amounts of annotation, such as named entities, are given for all sets, including the test set, and do not need to be predicted. If a node or edge in the graph format does not have the "given" attribute, it is considered a *target *and marked as such in the learned annotation scheme.

An example of a learned annotation scheme for the 2013 GENIA task is shown in Table 1. In learning the definitions described above, various types of attributes are required in the graph elements for the system to fully learn the annotation scheme. These attributes are automatically generated from the original Shared Task annotation, when converting from the BioNLP Shared Task format.

**Table 1:** The BioNLP'13 GE task annotation scheme, automatically learned from the corresponding corpus.

Type	Name	Arguments
ENTITY	Anaphora	
ENTITY	Entity	
ENTITY	Protein	

EVENT	Acetylation [2,2]	Site {Theme} [1,1] Entity / Theme [1,1] Protein
EVENT	Binding [1,4]	Site {Theme} [0,2] Entity / Theme [1,2] Protein
EVENT	Deacetylation [2,2]	Cause [0,1] Protein / Site {Theme} [0,1] Entity / Theme [1,1] Protein
EVENT	Gene expression [1,1]	Theme [1,1] Protein
EVENT	Localization [1,2]	Theme [1,1] Protein / ToLoc [0,1] Entity
EVENT	Negative regulation [1,3]	Cause [0,1] Acetylation, Binding, Gene expression, Negative regulation, Phosphorylation, Positive regulation, Protein, Protein catabolism, Regulation, Ubiquitination / Site {Cause,Theme} [0,1] Entity / Theme [1,1] Binding, Gene expression, Localization, Negative regulation, Phosphorylation, Positive regulation, Protein, Protein catabolism, Regulation, Transcription, Ubiquitination
EVENT	Phosphorylation [1,3]	Cause [0,1] Protein / Site {Theme} [0,1] Entity / Theme [1,1] Protein
EVENT	Positive regulation [1,3]	Cause [0,1] Acetylation, Binding, Gene expression, Negative regulation, Phosphorylation, Positive regulation, Protein, Protein catabolism, Regulation, Ubiquitination / Site {Cause,Theme} [0,1] Entity / Theme [1,1] Binding, Deacetylation, Gene expression, Localization, Negative regulation, Phosphorylation, Positive regulation, Protein, Protein catabolism, Protein modification, Regulation, Transcription, Ubiquitination
EVENT	Protein catabolism [1,1]	Theme [1,1] Protein
EVENT	Protein modification [1,1]	Theme [1,1] Protein
EVENT	Regulation [1,3]	Cause [0,1] Binding, Gene expression, Localization, Negative regulation, Phosphorylation, Positive regulation, Protein, Protein modification, Regulation / Site {Cause,Theme} [0,1] Entity / Theme [1,1] Binding, Gene expression, Localization, Negative regulation, Phosphorylation, Positive regulation, Protein, Protein catabolism, Protein modification, Regulation, Transcription
EVENT	Transcription [1,1]	Theme [1,1] Protein
EVENT	Ubiquitination [1,2]	Cause [0,1] Protein / Site {Theme} [0,1] Entity / Theme [1,1] Protein

RELATION	Coreference, directed	Subject(Anaphora) / Object(Anaphora, Entity, Protein)
RELATION	SiteParent, directed	Arg1(Entity) / Arg2(Protein)

MODIFIER	negation	Binding, Gene expression, Localization, Negative regulation, Phosphorylation, Positive regulation, Protein catabolism, Regulation, Transcription
MODIFIER	speculation	Binding, Gene expression, Localization, Negative regulation, Phosphorylation, Positive regulation, Protein catabolism, Regulation, Transcription, Ubiquitination

TARGET	ENTITY	Acetylation, Anaphora, Binding, Deacetylation, Entity, Gene expression, Localization, Negative regulation, Phosphorylation, Positive regulation, Protein catabolism, Protein modification, Regulation, Transcription, Ubiquitination
TARGET	INTERACTION	Cause, Coreference, Site, SiteParent, Theme, ToLoc

The *entities *form the nodes of the graph. The *events *and *relations *connect the nodes together and are defined by a type and a specific, limited set of arguments. Both event and relation arguments have specified valid target node types. Site-argument primary argument types are shown in wavy brackets. Event arguments have also a minimum and maximum amount of each argument allowed per event, and the event itself has a minimum and maximum number of arguments. The relations must always have two arguments and can optionally be directed. *Modifiers *are binary attributes that can be applied to a limited set of node types. The *targets *define the types of nodes and edges to be automatically extracted.

The learned annotation scheme is stored in the TEES model file and is available for the other program components which use it to enforce task-specific constraints in the machine learning steps. The most important use for the learned annotation scheme is in the edge and unmerging detectors where the rules can greatly reduce the number of examples that need to be classified. Especially the unmerging step would become computationally unfeasible without such filtering.

#### The impact of the learned annotation scheme on the TEES pipeline

In *edge detection*, the learned annotation scheme is used to limit edge example generation for the pairs of nodes between which a valid edge (either an event argument or a binary relation) can exist. This filtering greatly reduces the number of negative examples, for example in the 2013 GENIA task training set, edge example filtering removed 18,962 negative examples out of a total 25,802. With only 3,278 positive examples in the training set, this filtering produces a more balanced class distribution and greatly speeds up the machine learning by removing most of the examples that can ever only be negative.

In the *unmerging step*, each candidate event example is validated using the learned annotation scheme, removing all candidates that are not structurally valid events. The type of the trigger node, as well as the types and numbers of outgoing edges are considered, allowing the unmerging step to only predict as positives structurally valid events.

Before automated annotation scheme learning, these limits had to be manually written in the program code. This was reasonably doable for edge limits, as only lists of valid node pairs needed to be defined. However, the complexity of the event rules required in the unmerging step meant that the TEES system could only construct valid events for the small number of corpora for which these extensive rules had been written in the code. With the automated annotation scheme learning system not only the system can be transparently applied to any event corpus, but also no extension of the system through additional programming is needed. Thus, the automated annotation scheme learning for the first time enables the TEES system to be easily utilized on novel corpora outside the BioNLP Shared Tasks.

#### TEES 2.2 annotation scheme learning extensions

The automated annotation scheme learning system was developed to quickly adapt TEES to the new corpora introduced in the BioNLP'13 Shared Task. It mostly achieved this goal, but the learned scheme had a few limitations that on occasion resulted in incorrect output. In this paper, we address these limitations and enhance the annotation scheme learning to fully grasp all the details of the BioNLP event corpora.

In the CG13 Task the *Glycolysis *event never takes any arguments. As Glycolysis-nodes never had outgoing edges, the TEES 2.1 learning system defined Glycolysis as an entity, which caused issues when converted back to the Shared Task format. In TEES 2.2, all BioNLP Shared Task event trigger nodes have the "event" attribute, enabling the system to correctly learn that *Glycolysis *is an event even if it never has arguments.

In the TEES 2.1 version, used in the BioNLP'13 Shared Task, event definitions had a minimum and maximum number for each valid argument type, but no overall argument count limitations. After the Shared Task we speculated that errors could result from events with optional arguments of which at least one is required [11]. To overcome this limitation, the TEES 2.2 annotation scheme learning system defines also overall argument limitations for an event, so that for example an event that takes 0-1 arguments of type A and 0-1 arguments of type B can still be required to have at least one argument, total. In practice however such situations do not appear in the BioNLP'13 corpora, with all events that have only optional arguments also allowing events with no arguments.

A more important source of errors, resolved in the TEES 2.2 version, is issues related to Site-arguments, described in the next section.

### Unified Site-argument representation

The *Site*-arguments are one of the more complex parts of the BioNLP Shared Task annotation. These arguments add detail to other arguments, and by connecting a separate Site-entity to the primary argument define the substructure of the gene or protein that is the ultimate target of the argument. When straightforwardly converted to a graph representation a Site-argument would be an edge connected to another edge, a structure not possible to implement without the target edge being split in two by a redundant central node to which the site-argument could connect. Such an approach would necessitate a multi-stage edge detection system, as site-arguments could only be predicted when the primary arguments to which they connect have already been predicted.

**Figure 2:**
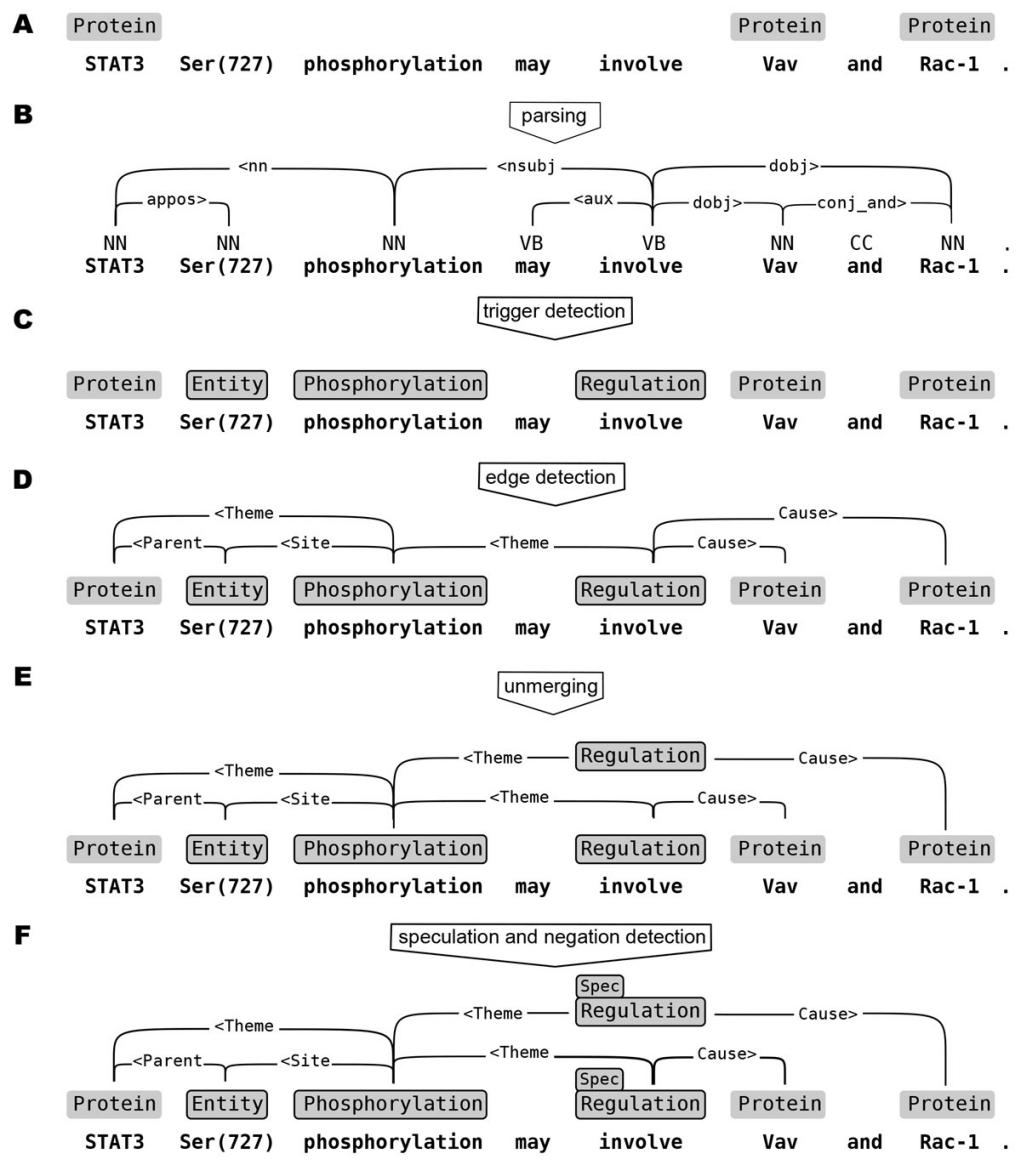
**Multiple approaches (A and B) were used in the BioNLP'11 Shared Task for representing site-arguments in the TEES graph format**. In TEES 2.0 these representations have been merged into the unified representation (C), allowing site-arguments to be processed like any other event arguments.

In developing the TEES system, several approaches have been tried to address the detection of site-arguments as part of general edge detection. In TEES 2.0 site arguments were defined as edges connecting a site entity to its parent protein (See Figure 2A) or to the trigger node (See Figure 2B). The second case (used in the 2011 EPI task [5]) was more straightforward and possibly closer to the syntactic structure, but could only be used for events that had a single primary argument and thus an unambiguous connection between the primary and the site argument. However, some ambiguity remains also in the first case (Figure 2A), because even when the connection between the protein and its site is clear, there may be multiple events only some of which refer to the site in addition to the protein. This inconsistency means that the automated annotation scheme learning system introduced in TEES 2.1 cannot learn the valid site argument constraints for events from such a graph.

Therefore, in TEES 2.1 a unified representation for site arguments (See Figure 2C) is used, where site arguments are linked to the trigger node regardless of task, allowing straightforward processing of learned event argument limitation rules. Separated *SiteParent *edges are added, connecting the site entity to its protein.

The TEES 2.1 Site argument representation still had a few limitations, which became apparent after the shared task. In the current 2.2 version Site argument processing is further refined, finally covering all the intricacies of the BioNLP Shared Task annotation scheme.

#### TEES 2.2 Site-argument processing

In the graph representation it is possible for one event to get two Site-arguments, both of which connect to different site-entities, which in turn connect to the same protein through SiteParent-relations. As the protein is the target of the primary argument, when the graph was interpreted as Shared Task events, both of the sites referred to the same primary argument. This situation was resolved by discarding further Site arguments if the primary argument already had one.

In another case, SiteParent-relations were predicted so that a single site-entity had multiple Protein entities as parents. When different primary arguments linked to these Proteins, a single Site argument could be mapped to multiple primary arguments, and due to an error in the program, caused a duplication of these primary arguments. This issue has been resolved by preventing a Site-argument from being mapped to more than one primary argument.

These solutions can lead to removal of Site-arguments, which in turn can lead to formation of an event which is identical to another, existing event. To remove such duplicates, considered invalid in the BioNLP Shared Task, a final filtering phase is added when converting to the Shared Task format, recursively removing duplicates until none exist.

#### Site-arguments and TEES 2.2 automated annotation scheme learning

The solutions in the previous sections resolved almost all the structural issues in predicted BioNLP Shared Task events. One issue could not be fixed without updating the graph format: In the PC13 task there exist *Phosphorylation *events where both the *Cause *and the *Theme *refer to the same protein, describing self-phosphorylation events such as "PDK1 can phosphorylate itself at Ser-241".

When the site "Ser-241" is further linked through a Site-argument, the SiteParent-edge connecting the site-entity to "PDK1" cannot differentiate between *Cause *and *Theme*. In PC13 however, only Theme can have a *Site*-argument.

To solve these cases, the edge elements in the TEES 2.2 graph format can now have a "siteOf" attribute which unambiguously identifies the primary argument of the *Site*-argument. This information can of course only be included in known, annotated data, but it is enough to allow the automated annotation scheme learning system to learn that e.g. a PC13 *Phosphorylation *event's *Site*-argument can only have a *Theme *argument as the primary argument, thus preventing the error on predicted events of this scheme.

The automatically learned annotation scheme is extended with known primary argument types for all *Site*-arguments, resolving this remaining issue and providing fully structurally correct BioNLP Shared Task predictions (See Table 1).

### Validating TEES 2.1 BioNLP'13 predictions

The TEES 2.2 system described in this paper addresses most of the shortcomings of the TEES 2.1 system applied in the BioNLP'13 Shared Task. As the Shared Task test set evaluation servers will reject the whole submission when any structurally invalid events are present, the errors resulting from the limitations of the 2.1 system had to be resolved manually when participating in the Shared Task. However, such errors could not be fixed by looking at the test set and then correcting the events that prevented the acceptance of the submission, as that would result in a *de facto *manual annotation of the test set and thus an information leak.

To solve this problem we therefore never looked at the document triggering the error, and used a simple, consistent approach to resolve invalid events rejected by the server. In cases where the server reported both an invalid argument as well as a missing argument in the same event, the invalid argument type was replaced with the missing one's type. If the server only reported an invalid argument, the argument was removed, and if this did not resolve the error, the entire event was removed. After these steps, all events pointing to removed events were also recursively removed.

In the current 2.2 version of TEES the issues causing these errors have been fixed in various stages of the pipeline, and the automated annotation scheme learning system has been extended to be able to fully depict all aspects of the BioNLP Shared Task annotation scheme, as discussed in the previous sections. TEES 2.2 predictions for all BioNLP'13 tasks were accepted by the test set evaluation server without errors.

### Public dataset

By the summer 2012 the TEES 2.0 system had been developed to the point where it could be potentially useful for other participants of the BioNLP 2013 Shared Task. However, since the program itself had to be extended with additional code for each new corpus, application for the 2013 corpora was no small task. To finally solve this limitation the automated annotation scheme learning system was developed, following the generalization approaches developed for the 2011 task and making them consistently usable for all the new corpora.

Even then, training the TEES system remained a computationally intensive and potentially error-prone task for people not familiar with the program. To ensure easy application of TEES for the 2013 task, predictions for the new corpora were published during the system development period. The analyses for the development sets were made available on February 26th, followed by publication of test set analyses on April 13th (during the test period). These datasets did not enjoy wide popularity and were downloaded only a few times.

Due to the complexity of the BioNLP Shared Task efficiently utilizing a separate set of predictions in another system may likely have been too time consuming to experiment with. Similar precalculated TEES predictions were published also for the DDIExtraction 2013 Shared Task, where the datasets were used more, quite possible because such analyses could more easily be integrated into the more straightforward binary relation extraction task [15,18].

### Event Visualization

The BioNLP Shared Task events can form complex, nested structures. Neither the original Shared Task format nor the TEES graph representation are that easy to read, especially when complex nesting leads to long chains of linked events. Understanding the predicted graphs is often instrumental in analyzing system errors and devising better event extraction approaches. Therefore, in TEES 2.2, an integrated visualization system is provided with the program.

**Figure 3:**
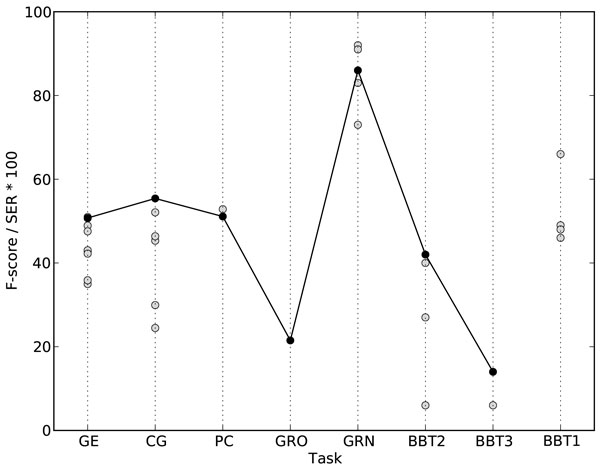
**The visualizer provided with TEES 2**.2 can be used to display both the event annotation as well as the parse of a sentence. This figure shows sentence GE13.d216.s0, taken from the BioNLP 2013 GENIA development corpus document PMC-3333881-20-Caption-Figure 3, demonstrating a nested event structure consisting of two *Negative regulation *events.

The TEES 2.2 visualizer relies on the open source graph drawing software GraphViz and its dot layout engine [19]. By giving a corpus file and the id of a sentence, the user can get an immediate visualization of the semantic annotations and syntactic parse of that sentence (See Figure 3).

The visualization is generated as a GraphViz dot language file, and rendered as a PDF file using the GraphViz program. By relying on the advanced graph layout algorithms of GraphViz, the TEES visualization tool remains simple but produces illustrative examples for even complex annotation graphs. The tool is intended to aid in system development and debugging, and if more consistent and higher quality visual representations are needed, a dedicated annotation visualization tool such as BRAT should rather be used [20].

### Integrating scikit-learn

Since the BioNLP 2009 Shared Task TEES has relied on the SVM*^multiclass ^*program for all classification tasks. In TEES 2.2 the interface to the classifier is generalized, allowing different classifiers to be plugged into the system.

Scikit-learn is an increasingly popular Python library for all kinds of machine learning tasks and provides also a wide selection of classifiers [21]. TEES 2.2 includes an interface for using scikit-learn, and in the results we introduce several examples of using different classifiers from this package. Scikit-learn classifier parameters can be defined through the TEES command line interface, and even simple Python structures, such as class weight dictionaries, can be passed to the system this way.

In addition to the Scikit-learn integration TEES 2.2 includes a dummy classifier, which simply returns the correct output for examples whose annotated class is known. This classifier can help in analyzing system performance on various tasks, and in this paper we use it to give an overview into the relative performance impact of the various learning components in the TEES pipeline.

## Results

### BioNLP'13 Shared Task

**Figure 4:**
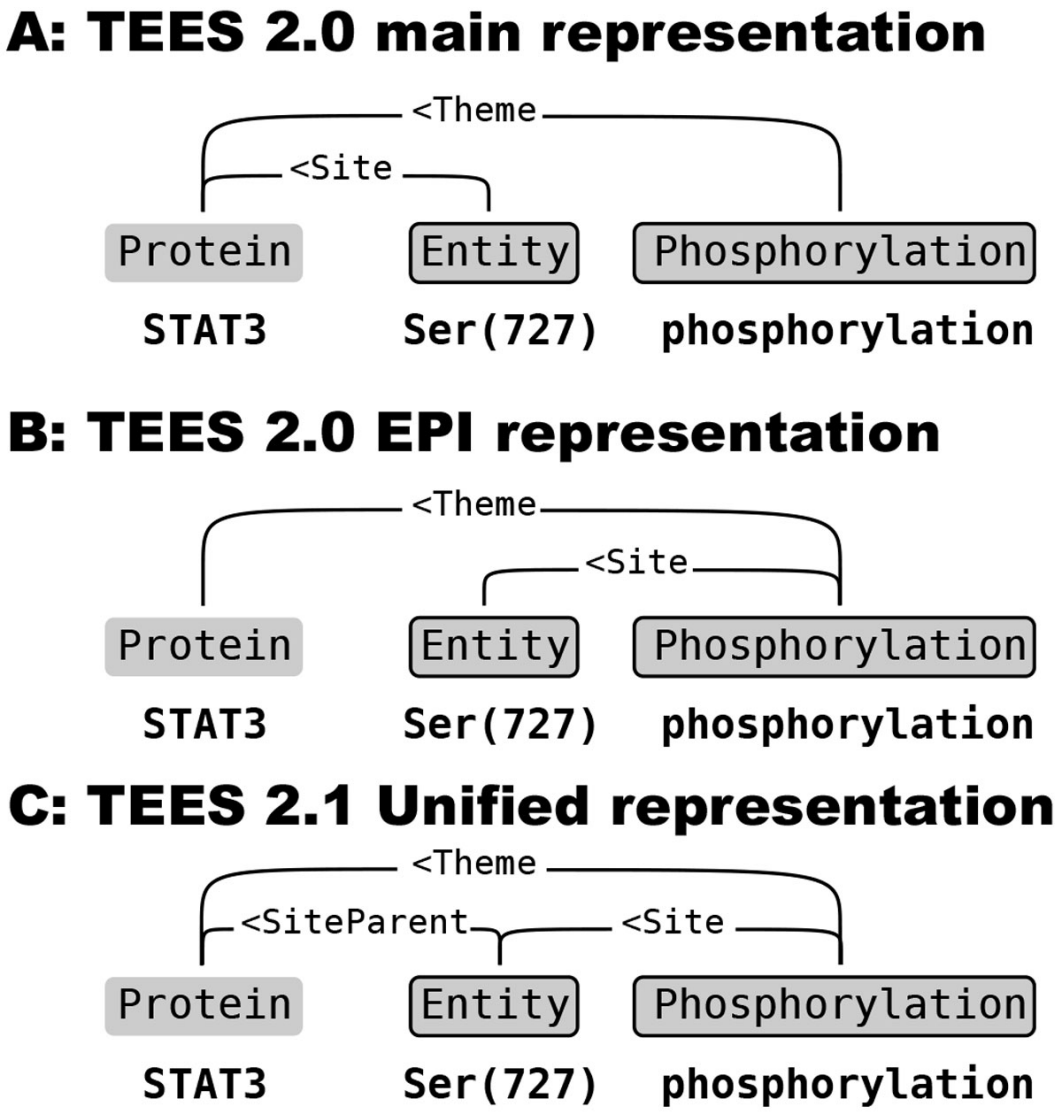
**The performance of systems that took part in the BioNLP'13 Shared Task**. The TEES results are shown with black crosses. Please note that in tasks GRN and BBT1 the metric is SER*100 where a smaller score is better.

Due to the automated annotation scheme learning, it was possible to apply TEES 2.1 to almost all the 2013 tasks with task specific development no longer required. Only the subtask 1 of the Bacteria Biotopes task, concerning boundary detection and labeling of entities with concepts from a large ontology, fell outside the scope of the TEES system. In the end, TEES 2.1 was the system that participated in most tasks and demonstrated generally good performance. These results confirmed the validity of abstracting away task-specific details in developing the system. The official results for the tasks of the BioNLP 2013 Shared Task are listed in Table 2 and the performance of the TEES 2.1 system relative to other entries is displayed in Figure 4.

**Table 2:** Official BioNLP 2013 Shared Task results for the TEES system showing performance on the hidden test sets.

Task	#	R	P	F	SER
GE13	2/10	46.17	56.32	50.74	
CG13	1/6	48.76	64.17	55.41	
PC13	2/2	47.15	55.78	51.10	
GRO13	1/1	15.22	36.58	21.50	
GRN13	3/5	33	78	46	0.86
BB13 T1	0/4				
BB13 T2	1/4	28	82	42	
BB13 T3	1/2	12	18	14	

The performance is defined by the (F)-score, composed of (R)ecall and (P)recision. The SER metric is used for the the GRN task. The BB task 1 is outside the scope of the TEES system. Placement among other systems is indicated by #.

#### GENIA (GE13)

Of all tasks in the BioNLP Shared Task the original GENIA task remains central and has been organized in all three iterations. This task has also been the most popular among participants and could therefore be viewed as the main platform for competitive evaluation of diverse event extraction approaches. While the 2009 and 2011 GENIA tasks were very similar, in 2013 the GENIA task annotation has been considerably extended and the co-reference annotation (a separate task in 2011) has been integrated in the GENIA corpus [22]. The advantage here is that supporting information like co-references is more likely to be utilized, but on the other hand direct comparability with the earlier iterations of the task is lost.

The GENIA task corpus is a good example of the benefits of automated learning of an event annotation scheme. The corpus is quite diverse, having 11 separate event types, a pairwise binary co-reference relation scheme and a modality annotation for negated and speculative events. In earlier TEES versions this whole scheme would have been encoded in the functionality of its example generation code, but the TEES 2.1 system can learn these rules automatically from the corpus data itself and store them in a re-usable, generalized structure information resource file. The automatically learned annotation scheme for the 2013 GENIA task is shown in Table 1. Such a scheme has been "reverse engineered" from the corpus, so it must be noted that it may not always correspond perfectly to the official annotation rules of a corpus. In particular, the *Binding *event can officially have one or more *Theme *arguments, but from looking only at the examples in the corpus itself, the automated system learns that two is the maximum number of *Theme *arguments.

For a subset of the co-reference relations in the GENIA corpus (45 out of 338 in train and devel data) one or both of its annotated endpoints are event trigger words. Such relations could be linked to the event trigger nodes in the TEES graph representation, but as this graph has no distinction between the trigger and the whole event, the system would connect these relations to the event annotation when converting back to the Shared Task format, so these relations were skipped.

In this 2013 GENIA task TEES reached second place at 50.74% F-score, with the first place achieved by team EVEX [23], who utilized the publicly available TEES 2.1 program. These good results show the benefits of utilizing available existing systems as part of new solutions and the value of open sourcing scientific code.

The third ranked system in the GE13 task with an F-score of 50.68% was the BioSEM by Bui et al. [24]. The BioSEM system uses shallow parsing and automatically learned syntactic event patterns that are matched against this parse. BioSEM is highly computationally efficient, reaching processing speeds of 3.4 ms per sentence whereas TEES and the 2011 Shared Task winning UMass system use 1040 and 1400 ms, respectively.

#### Cancer Genetics (CG13)

The CG task presents an event corpus developed for text mining related to cancer [25,26]. This corpus introduces a large variety of events and entities. Such diversity results in very many small classes in the TEES multi-class approach, but despite this potential issue, TEES 2.1 reached an F-score of 55.41% and a first place in the CG task. It is interesting to note that on some event categories TEES reached unusually high performance, for example an F-score of 77.20% for Anatomy-group events. It is interesting to speculate that the detailed annotation scheme of the CG corpus may have resulted in a very consistent annotation, allowing high machine-learning performance in this task.

#### Pathway Curation (PC13)

The PC task follows the database focus of the BioNLP'13 Shared Task, presenting a corpus of events designed to be applicable for pathway curation [27]. The PC events are based on known pathway annotation models and ontologies such as the Systems Biology Ontology (SBO). The PC corpus has only a few entity types but a large number of event types.

The TEES 2.1 system placed second in this task, 1.74 percentage points behind the NaCTeM team [28]. On the CG task team NaCTeM placed second, 3.32 percentage behind TEES 2.1. Thus, even when there were only two participants in the PC task, and these participants being very close in performance, we speculate that the PC corpus is of similar complexity with the CG corpus.

#### Gene Regulation Ontology (GRO13)

The aim of the GRO task is to evaluate the automatic annotation of texts with Gene Regulation Ontology (GRO) concepts [29]. For a BioNLP Shared Task task, the annotation is unusually detailed, consisting of 145 entity and 81 event types. This presents an extremely difficult situation for a classifier-based approach, with very many classes that have only a few examples each. Unsurprisingly, TEES could not detect most of the small classes and in general exhibited better performance the larger the class.

The 2011 EPI task had a number of small "reverse" event classes (such as *Dephosphorylation *for *Phosphorylation*). In many cases the reverse class could be combined to or separated from the main class with only a few simple rules, and this approach was successfully used in the TEES entry to make many small classes detectable. A similar approach could potentially be used with the GRO corpus but finding classes to combine and determining the rules for this would be no small task.

TEES 2.1 was unfortunately the only system to participate in the GRO task. The performance of 21.50% F-score is quite low, but with no points of comparison, not many conclusions can be drawn from it. Still, TEES did perform decently on the larger classes, so it is conceivable that with a larger training corpus the performance could be increased to similar levels as seen in other tasks such as GE, CG or PC.

#### Gene Regulation Network (GRN13)

The GRN task takes the most direct approach for measuring the applicability of extracted events [30]. The events and relations produced in this task are automatically converted to a regulation network, and it is even possible to compete by ignoring the event stage and directly producing this network. Either way, the quality of this network is the GRN task's performance metric, with the Slot Error Rate (SER) [31] used as the measure, where lower is better and a value of less than one is expected for decent predictions.

The GRN corpus itself is slightly different from the more event-focused corpora in other tasks. Its annotation scheme defines 11 entity types, 12 binary relation types and only a single event of type *Action*.

TEES 2.1 was used for producing the events, which were then converted by the organizers to the final regulation network. A SER of 0.86 was achieved, placing TEES in the middle of the five participating teams. All of the teams had an SER of less than one, indicating predictions of useful quality. The best result of 0.73 was achieved by the team from the University of Ljubljana who used linear chain conditional random fields combined with rule-based detection of events and relations [32].

The GRN task organizers also provided their downloadable evaluator program early enough in the development period that it could be integrated to the TEES 2.1 system, allowing optimization against the official metric of the task. As the SER metric had not been used in the context of TEES event extraction, we decided to use the relaxed F-score as the optimization metric, on the basis that it had been shown before to predict well the performance on the hidden test set. During the development phase we however observed that generally the optimal SER score correlated with the optimal relaxed F-score.

#### Bacteria Biotopes (BB13)

The BB task, introduced in 2011, is the only task apart from GENIA to continue from an earlier BioNLP Shared Task. The goal in the BB task is to detect statements concerning bacteria habitats and their environmental properties. The BB task is divided into three separate subtasks [33].

The goal in task 1 is detection of bacteria habitat entity boundaries. Further, each detected entity must be assigned one or more terms from the 1700 concepts of the OntoBiotope ontology. The BB task 1 is the only task for which the TEES 2.1 system was not applied. While TEES has been extended to detect multi-token entities (in the 2011 CO task) it is not optimized for this type of classification. More importantly, assignment of the 1700 concepts is a challenge not easily approachable with the classification-centric approaches in the TEES system, and as our focus was on developing a generalized event extraction system, we considered the completely different approaches required for the BB task 1 to fall outside the scope of our current system.

On the other hand the BB tasks 2 and 3 form a direct continuation for the BB task of 2011. In these tasks the goal is to detect bacteria, habitat and geographical place entities and the relations between them. The annotation is very concise, consisting of only three entity and two relation types. In task 2 the competitors are provided all entities, resulting in a straightforward relation detection task, but in task 3 also the entities must be predicted.

Of all the BioNLP'13 tasks, the BB tasks were the only ones in which limited task specific resources were used to enhance TEES performance, mostly because the TEES 2.0 resources from the 2011 BB task were directly applicable also for the current ones. As in 2011, a dictionary of bacteria name tokens derived from the List of Prokaryotic names with Standing in Nomenclature (http://www.bacterio.cict.fr/) [34] was used to improve entity detection performance, but unlike the 2011 task, the WordNet features were not used this time.

The TEES 2.1 system achieved first places in both the BB tasks 2 and 3. Despite this, the performances of 42% and 14% of F-score (for tasks 2 and 3 respectively) are still relatively low, demonstrating the complexity of these tasks.

### TEES 2.2 performance

The TEES system has been developed since the 2009 BioNLP Shared Task, and has participated in all three BioNLP Shared Tasks. While the basic pipeline design has remained the same, the system has also changed with improvements such as the introduction of the automated annotation scheme learning system. To measure how the current system compares to the versions used in the shared tasks, we show in Table 3 current performance and the results from the various tasks. The comparison shows that the current performance differs little from the results of the tasks, some having now a slightly higher and some a slightly lower performance. The larger changes in the EPI11 and ID11 tasks result from work following the 2011 Shared Task, discussed in [5]. Generally, it can be said the TEES system has mostly evolved in scope: While the original 2009 system was only capable of processing the 2009 corpus, and even then only as a series of manually managed independent programs, the current TEES 2.2 version can be applied to any of the Shared Task corpora, can be used by other research groups, and with the automated annotation scheme learning system can process also corpora outside the BioNLP Shared Tasks.

**Table 3:** Turku Event Extraction System in the BioNLP Shared Tasks.

Task	Name	devel / test	devel 2.2 / test 2.2
GE09 1	GENIA Event Extraction	- / 51.95	49.11 / -^a^
GE09 2	Protein Site Arguments	- / -	- / -^a^
GE09 3	Negation & Speculation	- / -	- / -^a^

GE11 1	GENIA Event Extraction	55.78 / 53.30	53.91 / 54.03
GE11 2	Protein Site Arguments	53.39 / 51.97	-^b^/-^b^
GE11 3	Negation & Speculation	38.34 / 26.86	37.92 / 31.85
EPI11	Epigenetics and PTM:s	56.41 / 53.33	60.03 / 56.22
ID11	Infectious Diseases	44.92 / 42.57	50.56 / 49.96
BB11	Bacteria Biotopes	27.01 / 26	30.87 / -^a^
BI11	Bacteria Gene Interactions	77.24 / 77	76.81 / -^a^
CO11	Protein/Gene Coreference	36.22 / 23.77	30.11 / -^a^
REL11	Entity Relations	65.99 / 57.7	-/-^a^
REN11	Bacteria Gene Renaming	84.62 / 87.0	85.04 / -^a^

GE13	GENIA Event Extraction	51.43* / 50.74	50.13* / 49.18
CG13	Cancer Genetics	61.82* / 55.41	63.50* / 54.99
PC13	Pathway Curation	57.63* / 51.10	59.74* / 49.90
GRO13	Gene Regulation Ontology	47.18* / 21.50	47.42* / -^a^
GRN13	Gene Regulation Network	-^c ^/ 0.86 SER	-^c ^/ 0.85 SER
BB13 1	NER and Categorization	-/-	-/-
BB13 2	Bacteria Localization	11.81* / 42	13.71* / 42.20
BB13 3	Bacteria Entities & Relations	64.67* / 14	63.34* / 14.24

The first results column shows the official competition results from each BioNLP Shared Task in which TEES participated, reflective of the system's performance at that point in its development. The second column shows the performance of the current TEES 2.2 system. The development set results are evaluated using an official downloadable evaluator or the TEES internal evaluator (the latter shown with a star). The test set results are the results from the shared task (first column) or evaluated with the official online evaluation service (second column). The superscript (a) indicates that the online evaluation service is no longer available, (b) that that competition metric is not provided by the online evaluation service and (c) that the downloadable evaluator failed to process the predicted events.

A disturbing aspect of the evaluation is that many of the online test set evaluation services are no longer available. Five years after the original BioNLP'09 Shared Task the test set of that task can no longer be evaluated, and three years after the BioNLP'11 Shared Task only three out of the eight task test set evaluation services remain available.

### System component performance

By replacing one or several of the TEES pipeline components with an "always correct" dummy classifier that simply gives the examples the known, correct class, the performance of the components relative to the overall system can be evaluated. By replacing a single component with the "always correct" classifier, we can get an estimate of how much there could be to gain from focusing development on this component. By replacing all classifiers in the pipeline until a certain step with the "always correct" classifier, we can get an estimate of how much of the system error is due to this step. This evaluation resembles the one performed on an earlier version of TEES used in the BioNLP'09 Shared Task [4]. We perform this evaluation on the GENIA 2011 corpus, as it is a representative corpus for the BioNLP Shared Tasks and has a downloadable evaluator available.

The results are shown in Table 4. Using correct results for all steps gives an estimate of theoretical maximum system performance. At 95.99% F-score this indicates the impact of ignoring sentence boundary crossing interactions.

**Table 4:** System component performance in F-score evaluated by replacing individual processing steps with an "All Correct" classifier that always returns the correct result.

All Correct	Simple	Binding	Regulation	All
None	76.87	50.44	42.87	55.99

Entity	93.17	65.72	65.37	75.32
Edge	89.70	71.63	72.46	78.62
Unmerging	86.53	77.30	61.07	72.76

Entity + Edge	98.40	77.99	93.38	93.32

All	98.40	94.63	94.75	95.99

The left column shows the step or steps replaced. The other columns show performance for GE11 event subsets, where *Simple *refers to the five single-argument event types and *Regulation *to the three regulation types. Performance is evaluated for task 1 on the development set with the downloadable GE11 evaluator.

Replacing a single classifier at a time, the use of all correct results for the entity detection step achieves the highest improvement on simple (single-argument) event detection. This indicates that the detection of such events is mostly dependent on the correct trigger. Somewhat surprisingly, all correct results for the unmerging step give a considerably large improvement to Binding event extraction performance, indicating that the entity and edge detection steps do correctly detect Binding event components, but these are not always correctly combined into full events.

By replacing both entity and edge predictions with correct results, system performance is at 93.32%, only 2.67 percentage points below the result with also unmerging predictions correct. This shows that only a minority of events depend on the unmerging step, but as seen from the result with Binding events mentioned in the previous paragraph, the unmerging step can also compensate for errors made by earlier steps.

### Using scikit-learn classifiers

The new generalized classifier system allows using external classifiers, such as those of the scikit-learn system with TEES. While technically any scikit-learn classifier could be used, in practice only those supporting sparse feature matrices can be used, due to the large number of features and examples TEES produces.

To give a short overview of the variety of new classifiers available through scikit-learn, we show in Table 5 the impact of replacing the SVM*^multiclass ^*classifier with various scikit-learn classifiers. The LibSVM (SVC) and LibLinear (LinearSVC) classifiers used through scikit-learn unsurprisingly demonstrate good performance. With SVC, the default RBF kernel was used. With LinearSVC the decision function method was used to determine classification confidence scores. As the SVC classifier uses a one-vs-one classification, per-class confidence scores are not directly available, so class membership probability estimates are used instead [35]. Both the LinearSVC and SVC classifiers show a slight increase in performance (1.43 pp and 1.04 pp) over the SVM*^multiclass ^*classifier.

**Table 5:** GE11 event extraction with scikit-learn classifiers.

Classifier	Parameters	Recall	Precision	F-score
BernoulliNB	alpha = 0.001,0.01,0.1,1,10,100,1000	53.41	14.93	23.34
Perceptron	default	38.82	61.73	47.67
SVC	C=[10^-3^, 10^6^], probability=True	47.06	66.05	54.96
LinearSVC	C=[10^-3^, 10^6^]	46.65	68.02	55.35
ExtraTrees	n_estimators = 10,50,100	27.97	78.58	41.25
RandomForest	n_estimators = 10,50,100,500	24.92	78.65	37.84

SVM*^multiclass^*	C=[1,10^6^]	54.98	52.89	53.92

The SVM*^multiclass ^*used in all TEES BioNLP Shared Task results is shown for reference. Performance is evaluated for task 1 on the development set with the downloadable GE11 evaluator.

The simple Naive Bayes classifier for multivariate Bernoulli models (BernoulliNB) has low overall performance, but decent recall. The linear Perceptron classifier has somewhat better performance, but reaches good precision at the cost of recall.

With scikit-learn version 16 tree-based systems support also sparse matrices. We tested the Random Forest and Extra Trees ensemble methods which have generally good performance on a variety of tasks. However, in this task performance was considerably lower than with an SVM and despite the classifiers' ability to handle the sparse datasets, the memory consumption and processing times were excessive. However, these ensemble methods provide an internal ranking of feature importances, providing the opportunity to analyze the huge feature sets used by TEES in more detail (see Section Analyzing TEES features).

Not too many conclusions can be drawn from the evaluated scikit-learn classifiers. The good performance of the various SVMs is to be expected, and while the simpler methods also have unsurprisingly moderate performance, it has to be remembered that the current TEES system has been tuned for use with an SVM classifier since 2009. The most important result of integrating scikit-learn is that with an actively developed machine learning library, the suitability of new classifiers for event extraction can quickly be tested as they become available.

### Analyzing TEES features

The scikit-learn ensemble methods provide an estimate of features importances, with relative weights given for all features used by the system. These classifiers implement a measure known as "gini importance" or "mean decrease impurity", defined as the total decrease in node impurity averaged over all trees of the ensemble [36]. We use the ExtraTreesClassifier as the ensemble method for determining the feature importances. Even if its classification performance is not on par with the SVM the TEES system was developed with, it still uses the exact same feature sets which are the topic of this analysis.

**Figure 5:**
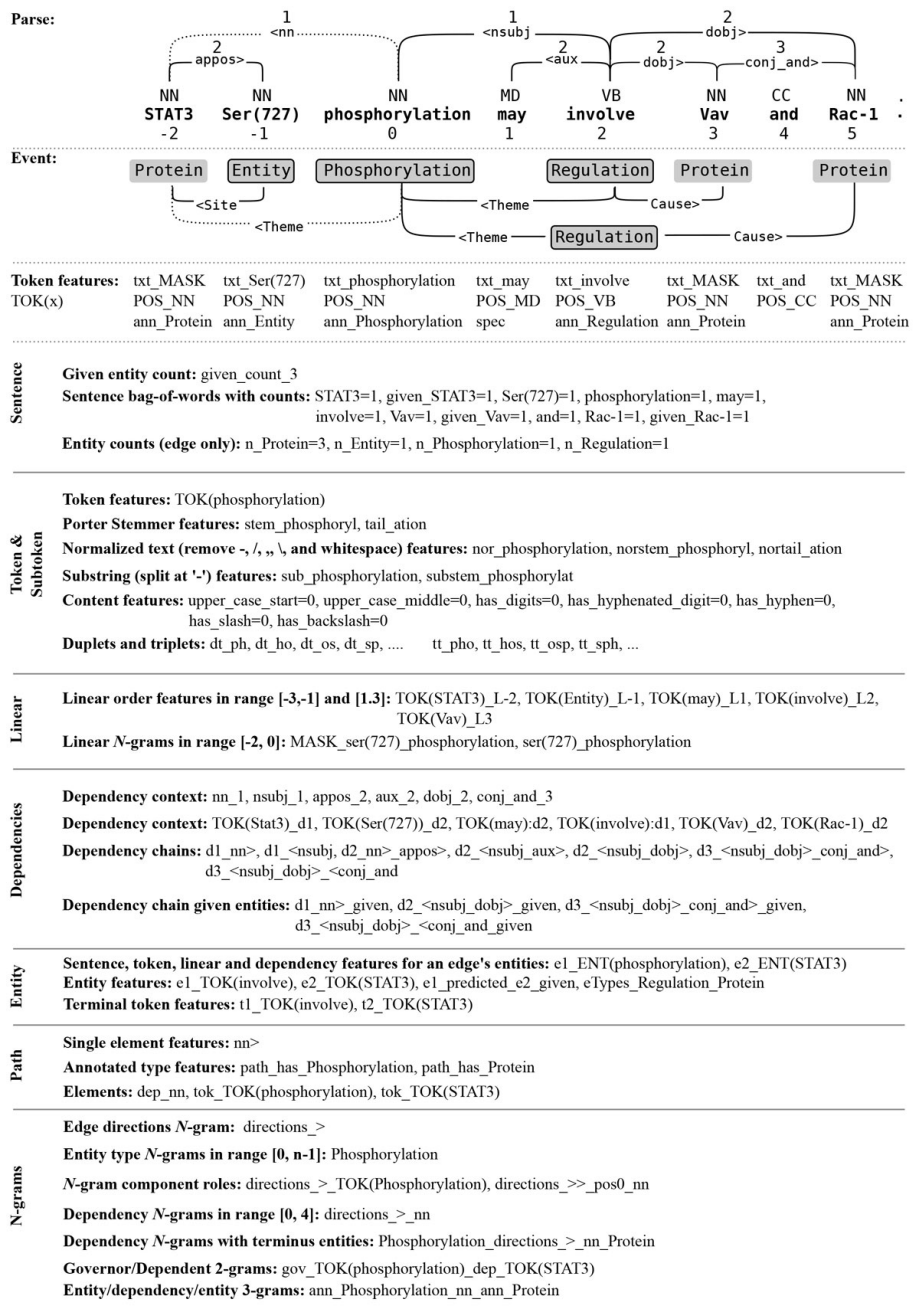
**Examples for the feature groups in Figure 6 and Table 6**. The numbered dependencies and tokens indicate the *linear *and *dependency *context for the token "phosphorylation". The dotted Theme edge and its corresponding dependency indicate the shortest path of an event argument edge. The example features correspond to the "phosphorylation" entity and the dotted edge. The token features TOK(x) are incorporated into the more complex features. (Figure adapted from [13].)

The TEES features are produced by two primary feature generation systems. The entity example builder generates features describing a single word and its context in the sentence. The edge example builder generates features from the shortest path of dependencies connecting two entities and their context in the sentence. The output of these generators can be further divided into groups, such as the *dependency context *of a node or *n-grams *built from the shortest path. These entity and edge example builders are used in various combinations to build the feature sets for the four classification steps (*trigger, edge, unmerging *and *modifier *) used in TEES. Examples of the features and feature groups used in TEES are shown in Figure 5. For a detailed discussion of the TEES feature representations we refer to Chapter 3 of [13]. For an analysis of the feature sets of the original TEES 1.0 system please see the analysis using the accumulated effect evaluation (AEE) algorithm of Xia et al. [37].

#### Feature Groups

**Figure 6:**
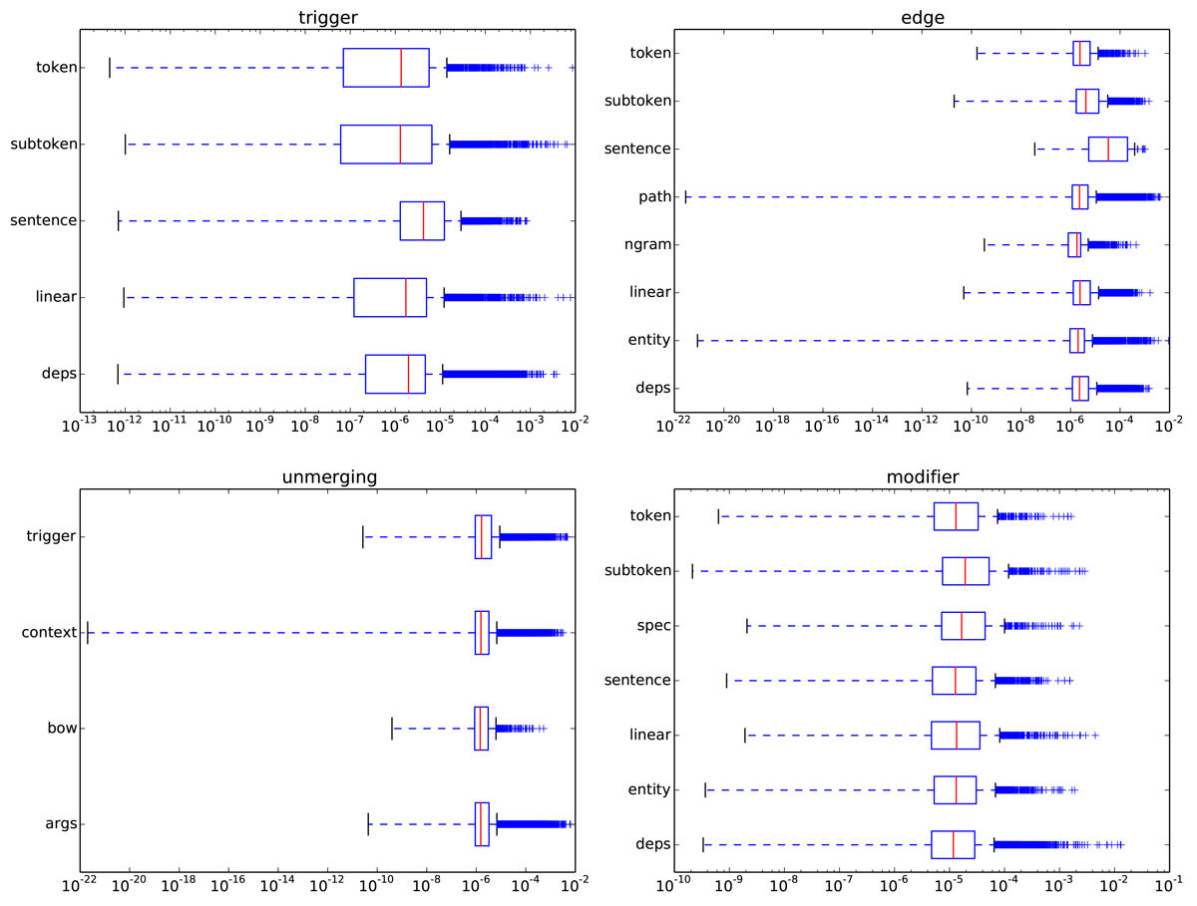
**The distribution of feature importances for feature groups, for each of the four classification steps (*trigger, edge, unmerging *and *modifier *)**. The *deps *group refers to *dependencies*. In the box plots the boxes contain the features from the lower to upper quartiles, with a red line at the median. The dotted-line whiskers extend to 1.5 times the interquartile range and the outlier points are shown as individual markers. See Figure 5 for feature group details.

Figure 6 shows the distribution of the feature importances for major feature types. Generally, no single feature group dominates the results, and all of the most important features are outliers. This indicates that even if feature selection can be used to increase performance, it is unlikely that the system could be optimized by removal of any of the feature groups, as important features exist in all groups. On a general level, Xia et al. report similar findings in their AEE feature selection experiment, although interestingly they also demonstrate a gain of 0.13 pp for TEES 1.0 trigger detection when discarding the dependency context features [37].

It is not surprising that *sentence *features are important for trigger detection, as these contain information about the presence of named entities and an overall picture of the content of the sentence. As such they could conceivably work as a very strong "on/off" switch when classifying the words of a sentence. However, it is surprising to see this same *sentence *feature group rank highly also among the edge detection features, which one would assume are more focused on the dependencies linking the two candidate entities. Predictably the *path *group, describing the overall structure of the shortest path has many important features for edge detection. The *n-grams *being generally less important than *path *features could be considered to result from them often describing longer structures and as such being less generally applicable.

The unmerging features consist of different combinations of the edge and trigger features. As such they are grouped according to this higher level of division. The *args *and *context *features describe the dependency paths corresponding to and outside of the event's arguments, respectively. The *trigger *group encompasses the feature groups of the event's trigger node and the *bow *group is a bag-of-words defined for the linear span of the event. The unmerging feature groups are rather equal in importance, with the exception of the *context *group containing very unimportant features, as is likely for the areas of the parse outside the candidate event.

The modifier detector uses an adapted version of the trigger feature builder. Its most important distinction is the *spec *group of features based on a manually curated set of known speculation-related words. That the dependency context *deps *ranks so highly for modifier detection can be explained by the fact that speculation and negation are largely expressed by modifiers connected to the trigger word token (e.g. "not phosphorylated" or "potentially regulates").

After this overall analysis of of the TEES feature groups we look in more detail at the individual, important features.

#### Top Features

As the feature group analysis (Figure 6) shows, important features can come from almost any feature group, regardless of the overall importance of said group. In Table 6 we list the features ranked as the most important for the four classification steps in TEES.

**Table 6:** Most important features as determined by the scikit-learn ExtraTreesClassifier.

step	#	weight	feature	group
trigger	1	0.0087	POS VB	token
	2	0.0078	linear_3_txt_I	linear
	3	0.0066	stem_induct	subtoken
	4	0.006	dt_on	subtoken
	5	0.0054	linear_3_txt_we	linear
	6	0.0054	linear_3_txt_was	linear
	7	0.0041	linear_-1_txt_inhibits	linear
	8	0.0041	dt_si	subtoken
	9	0.0038	dist_3_annType_Protein	dependencies
	10	0.0034	dt_xp	subtoken

edge	1	0.009	e2_txt_Id1	entity
	2	0.0042	tok_FFtxt_phosphorylation	path
	3	0.0039	dep_Reverse_dobj	path
	4	0.0036	tokenPath_Positive_regulation_e1_Positive_regulation_	path
	5	0.0035	GENIA_target_protein	entity
	6	0.0034	POS_VBZ	path
	7	0.0034	tok_RFFFtxt_mRNA	path
	8	0.0028	tok_RFFtxt_phosphorylation	path
	9	0.0025	tok_RRtxt_Id2	path
	10	0.0025	txt_block	path

unmerging	1	0.0064	argTheme_dep_Reverse_prep_of	args
	2	0.0062	argTheme_POS_NN	args
	3	0.006	argTheme_txt_expression	args
	4	0.0048	trg_dt_up	trigger
	5	0.0047	trg_chain_dist_dist_2-rev_appos-rev_punct	trigger
	6	0.0045	trg_dt_xp	trigger
	7	0.0043	trg_tt_ssi	trigger
	8	0.0041	argTheme_txt_affected	args
	9	0.0041	trg_dt_ex	trigger
	10	0.0041	argThemetrg_dep_dist_dist_3dep	args

modifier	1	0.013	t1HOut_neg_RB	dependencies
	2	0.013	t1HOut_neg	dependencies
	3	0.011	t1HOut_nsubjpass_NAMED_ENT	dependencies
	4	0.0089	dep_dist_dist_3neg	dependencies
	5	0.0074	t1HOut_not	dependencies
	6	0.0072	dist_3_txt_not	dependencies
	7	0.0053	dist_3_txt_significantly	dependencies
	8	0.0048	chain_dist_dist_1-rev_nsubjpass-frw_conj_and-rev_dep	dependencies
	9	0.0044	linear_-2_txt_was	linear
	10	0.0032	t1HOut_advmod	dependencies

See Figure 5 for feature group details.

The weights are relative for each classification step.

The trigger feature with the highest weight is unsurprisingly the VB part-of-speech label, as after all, trigger words are generally verbs. *Subtoken *features are also important, such as the common "induction" trigger word and the two-letter duplet "xp" most likely correlating with various forms of the word "expression". Trigger words are most often linked to named entities, so the presence of a *dependency *context feature "dist 3 annType Protein" is to be expected. Less clear are the high weights given to *linear *context features but words like "we" and "was" might indicate statements where experimental work is described (as in "we analyzed the phosphorylation. . . ").

While the edge feature groups show no clear winner, the top 10 features are unambiguous: The *entities *and the dependency *path *of the candidate edge are the most important features. The most important path features consist largely of individual words and their relative positions on the shortest path.

The unmerging features with the highest weights come from two groups: The *trigger *features of the event's trigger node and the edge features corresponding to the predicted *arguments *of the candidate event. Features for the dependency *context *outside the event itself are not among the highest weighted ones, suggesting that the entity itself, not its context in the sentence, is the most important basis for classification at this stage.

The most highly ranked modifier features confirm our speculation from the feature group analysis: The most important features for modifier detection are indeed the dependency types and words that describe a modification of the primary trigger word. While the modifier detection system detects both negation and speculation, among the top 10 features only one, the feature "dist 3 txt significantly", is related to a speculative context.

## Conclusions

### TEES 2.1 in the BioNLP'13 Shared Task

The TEES 2.1 system was successfully utilized in the BioNLP 2013 Shared Task. The automated annotation scheme learning system simplified considerably the application of the system to the diverse corpora, while still resulting in good general performance, reaching multiple first places.

The GRO task brought to light the limitations of the TEES approach when the annotation contains very many small classes. A similar situation made it unfeasible to apply the system to the BB task 1. Even with these limitations, the fact that the TEES system could be applied to most tasks with good results demonstrates that a basic stepwise SVM approach remains very much state-of-the-art in terms of predictive performance. However, such a generalized machine learning approach relying on massive feature sets is also computationally intensive, and it is refreshing to see novel, more specialized systems such as the BioSEM in the GE task demonstrating both computational performance and good prediction quality [24].

We continued our commitment to open source development by making the TEES 2.1 system publicly available already during the BioNLP'13 Shared Task's development phase, and provided precalculated analyses to all participants. While it is unfortunate that not many participants utilized these resources, the good results in the GE task, and also the performance demonstrated by system combination in the earlier BioNLP Shared Tasks [38,8] are convincing demonstrations of the value of merging together the areas of strength from several diverse systems.

TEES 2.1 demonstrated good performance on many of the BioNLP'13 Shared Tasks, but it must be considered that as a system that has participated also in the 2009 and 2011 tasks it had already the capacity to handle much of the basic work required to get started in event extraction. A particular advantage may have been the TEES internal micro-averaged F-score evaluation which provides a good approximation of the official metrics of many tasks. It is unfortunate that in the BioNLP'13 tasks official evaluator programs were often not available or published so late in the development period that they were unlikely to have much impact in guiding the teams towards the correct optimization goals. We consider it to be highly important that in shared tasks like this the official evaluation metric is clearly known well ahead of time, and also that the evaluation programs are made publicly available in order to avoid critical errors arising from multiple teams re-implementing the often complex evaluation methodology on their own.

### The future of past BioNLP Shared Tasks

A worrying aspect of the evaluation of current TEES 2.2 performance on older BioNLP Shared Task corpora was that so many of the test set evaluation services were no longer available online. Some are down due to moving servers, and while no doubt they will eventually be restored, the current shortage highlights an important concern about the long-term availability of scientific web services. The limits of web services have been analyzed e.g. by Schultheiss et al. [39].

The test sets of BioNLP Shared Tasks have been kept hidden to enable objective evaluation of new systems on older corpora. As one cannot expect the evaluation servers to be maintained indefinitely, it would perhaps be good if the older test set annotations were made publicly available, even if the latest round is kept as a hidden, objective resource. A public dataset can be distributed through different venues, ensuring its preservation even after the original source may be gone.

The BioNLP corpora represent some of the largest, most thoroughly annotated corpora in the field and it would be unfortunate if some of them would be lost to history.

### TEES 2.2 improvements

The automated annotation scheme learning system, introduced in TEES 2.1, has now been improved to produce fully structurally correct events for all BioNLP Shared Task corpora. These improvements should also make the TEES system more robust when applied to other, novel corpora.

The Site-arguments, which modify primary arguments, have been the subject of extensive system development when applying TEES to the BioNLP Shared Tasks.

One can wonder whether solving such a minor technical issue has been worth the efforts dedicated to it, considering how rare the ambiguous cases are. However, in the BioNLP 2013 Shared Task structural correctness of predicted events has been given extreme importance, to the extent that the evaluation servers refuse to provide any results unless all predictions are perfectly correct structurally. As manually resolving such errors is extremely tedious, complying with these structural requirements is a necessary step in ensuring further development on these corpora can proceed efficiently.

We introduced a new, general system for using different classifier tools within the TEES processing pipeline. The integration of the scikit-learn library provides access to numerous cutting edge classifiers, allowing the latest machine learning advances to be quickly applied to event extraction. The high dimensionality of text mining features still presents some challenges when selecting classifiers, but as the scikit-learn project evolves, more and more solutions are likely to become available also for event extraction.

As an example of the new directions enabled by the scikit-learn integration we performed a detailed analysis of the feature importances of the TEES system. This is the first time we have analyzed the huge TEES feature spaces outside trial-and-error system development. It is interesting to see assumptions made during the system development both supported and contradicted by the experimental analysis. The results of feature group analysis indicate that no single feature type is more important than the others, and that important features can arise from each group. This supports our experience that large, diverse feature sets provide a good basis for classification in event extraction.

### Program availability

The TEES system is a free and open source project. As with previous versions the current 2.2 series is publicly available from the repository (http://jbjorne.github.io/TEES/).

## Competing interests

The authors declare that they have no competing interests.

## Authors' contributions

JB designed and implemented the experiments. TS supervised the work.

## Declarations

Publication costs for this article were funded by University of Turku.

This article has been published as part of *BMC Bioinformatics *Volume 16 Supplement 16, 2015: BioNLP Shared Task 2013: Part 2. The full contents of the supplement are available online at http://www.biomedcentral.com/bmcbioinformatics/supplements/16/S16.
